# Positive and negative experiences with the COVID-19 pandemic among lonely and non-lonely populations in Germany

**DOI:** 10.3389/fpubh.2022.1067038

**Published:** 2023-03-02

**Authors:** Luisa Wegner, Shuyan Liu

**Affiliations:** ^1^Department of Education and Psychology, Freie Universität Berlin, Berlin, Germany; ^2^Department of Psychiatry and Psychotherapy, Charité – Universitätsmedizin Berlin (Campus Charité Mitte), Berlin, Germany

**Keywords:** social isolation, epidemic, environmentally friendly, perception, positive psychology, attribution bias, conspiracy beliefs

## Abstract

The COVID-19 pandemic is causing an epidemic of loneliness. Previous studies have shown the differences in positive and negative experiences of lonely and non-lonely people in a non-pandemic setting. However, it is unclear how the drastic alteration of the COVID-19 pandemic may influence peoples' reactions and beliefs, especially among those who feel lonely. Our study aims to examine the positive and negative experiences among lonely and non-lonely people. We undertook a cross-sectional online survey of the general population in Germany (*N* = 1,758) from May 2020 to May 2022. We assessed their feelings of loneliness with the short eight-item UCLA Loneliness Scale (ULS-8), their positive and negative experience of living in the COVID-19 pandemic as well as their psychological distress regarding the pandemic with the COVID-19 Peritraumatic Distress Index (CPDI). We found lonely individuals (ULS-8 score ≥ 16) reported fewer positive experiences of living in the COVID-19 pandemic, for example, less time with loved ones [*z*_(1, 756)_ = −2.5, *p* = 0.012] and less sense of togetherness [*z*_(1, 756)_ = −2.39, *p* = 0.017] as compared to non-lonely individuals. Meanwhile, they experienced more negative experiences, for example, worry and fear [*z*_(1, 756)_ = 6.31, *p* < 0.001] compared with non-lonely individuals. Interestingly, lonely people were less likely to view the pandemic as a conspiracy than non-lonely people were [*z*_(1, 756)_ = −3.35, *p* < 0.001]. Our results may give insight into attribution bias and the negative affect of lonely people during the COVID-19 pandemic as well as highlight the experience of non-lonely people and raise the question of differences in conspiracy beliefs. For pandemic preparedness and response, decision-makers may focus on interventions to foster social cohesion, empower people, build resilience, and most importantly provide timely social care.

## 1. Introduction

With the start of lockdown during the beginning of COVID-19, it was uncertain how long the significant changes that governments made would last. Those changes brought grand challenges to life, such as preventing the virus from spreading around the world (e.g., wearing a mask) (1), staying at home to protect children and vulnerable adults (2–4), and staying connected when physically apart (5–7). The World Health Organization reported that the global prevalence of anxiety and depression increased by a massive 25% in the first year of COVID-19 (8). Beyond health challenges, COVID-19 has also exacerbated social challenges (9–14), including attitudes toward social roles (e.g., gender roles) (15). However, dealing with this adversity is a chance to bring forward positive aspects as well. Wong's Existential Positive Psychology theory states that the great adversity that exists in life is a chance to bring forward positive experiences, indicating that suffering can promote strength and wellbeing (16). Wong et al. proposed that faith, courage, creativity, and the meaning of life are essential to transform suffering into flourishing (17). Existential positive psychology seeks to integrate various ways that help people experience positive states in times of suffering (17). To prepare for and respond to future pandemics, it is crucial to reflect on both positive and negative experiences that we had during the ongoing COVID-19 pandemic. When looking back, the COVID-19 movement restrictions did have a positive impact on the living environment (i.e., reduction in air pollution and emission of greenhouse gases) (7). In addition to making life more environmentally friendly compared to before COVID-19, the adaption to the new way of living (e.g., spending more time at home) provided opportunities for introspection (18). For example, people can for once rethink what is really important in their life, how they truly want to spend their time, and what they value most (18). This is a unique opportunity to generate ideas as well as to evaluate one's life. Moreover, staying at home during the lockdown enabled people to spend more time with loved ones (i.e., family members that live together in lockdown) (19). Another positive outcome of the unexpected COVID-19 lockdown was more time for hobbies (20). People chose hobbies that can be done in solidarity like spending time in nature and engaging in creative activities (20). Finally, working from home has advantages, such as greater work control and an improved work–life balance (21), which may lead to less work-related stress.

Despite the potential positive experiences of COVID-19, it is important to understand perceived negative experiences as well (22). Existential positive psychology suggests that negative experiences can add meaning to life by striving to overcome them (23). Considerable studies across countries reported negative effects of COVID-19 on mental health, including feeling worried, anxious, restricted, lonely, angry, depressed, and having sleep problems (24–29). People experience negative cognitive states, such as COVID-19-related worry, which can be an indicator of negative mood (26, 30). Moreover, such negative feelings or experiences are most common among vulnerable populations (e.g., the elderly, pregnant women and their children, people with disabilities, and chronic long-term health conditions) (2, 31). In addition, perceiving COVID-19 as more dangerous than seasonal influenza (32) was associated with a heightened perception of worry and fear (33). Interestingly, many people even believed the whole pandemic was a hoax and made up of secret organizations, possibly to change the “world order,” which is a narrative deeply rooted in antisemitism (34).

Strikingly, loneliness played a key role in triggering stress-related behaviors and cognition during lockdown (35). Even before COVID-19, loneliness was described as a “behavioral epidemic” in the population (36). During COVID-19, physical distancing can worsen this situation, as positive social contact is a key factor in battling loneliness (37). Other factors that can increase feelings of loneliness and may have been intensified under COVID-19 are living alone, infrequent contact with family or friends, dissatisfaction with living circumstances, chronic work and/or social stress, small social network as well as a poor quality social network, and marital or family conflict (38). A representative study from Germany showed that the prevalence of loneliness increased from 11% before COVID-19 to 27% during COVID-19 (37). Our recent study showed that up to 66% of people in Germany sometimes or always felt lonely amid COVID-19 (39). Furthermore, loneliness increased with a small effect size in the population that reinforces a “loneliness epidemic” (40). Similar results have been reported in the association between increased prevalence of loneliness, depression, and anxiety (41, 42).

The Evolutionary Theory of Loneliness (ETLs) conceptualizes that loneliness has an adaptive and non-conscious function of a person perceiving the world as more threatening (43). Furthermore, if people feel no sense of solidarity, it leads to them operating in a “selfish” mode, which can help them protect themselves in a potentially dangerous situation (43). This state is extremely aversive and thus is conceptualized to bring people to renew their social connections (38) and to protect themselves from being alone in a hostile environment (43). Considering these points, it highlights that lonely individuals may perceive an objectively neutral situation as more hostile than non-lonely individuals may. That raises an interesting question of whether lonely and non-lonely people react differently in the ongoing COVID-19 pandemic. Spithoven et al. highlighted that lonely people process information differently from non-lonely individuals (44). Lonely individuals interpret information with more negative expectations and have a hostile attribution bias and negative evaluation of self and others (44). They also tend to have a higher sensibility for socially threatening and socially negative stimuli (44). These cognitive biases tend to reinforce and strengthen counterproductive social behaviors (44, 45). These findings undermine the fact that in a socially shifting situation like a lockdown, lonely people may perceive the world differently from non-lonely people. One of the popular beliefs amid COVID-19 was conspiracy theories. There were mixed results on loneliness relating to belief in conspiracy theories. On the one hand, the induction of loneliness increases paranoid ideation (46) that correlated with COVID-19 conspiracy beliefs (47). On the other hand, instead of loneliness, people who endorse conspiracy theories are influenced by their personal willingness to conspire (48) and by social contagion through conventional and social media (49). The inconsistent results are encouraging a better understanding of the role of loneliness in conspiracy endorsement.

While there is a surfeit of studies measuring the negative experiences among lonely people amid COVID-19 (2, 8–14, 26, 31, 35), there is a lack of studies on positive outcomes. To better prepare for and respond to the future pandemic, it is crucial to understand both positive and negative lessons we have learned during the COVID-19 pandemic. In this study, we aim to examine the positive and negative experiences during COVID-19 among lonely and non-lonely individuals in Germany. We expect that there will be different positive and negative experiences of living in COVID-19 among lonely and non-lonely people. Specifically, we hypothesize that lonely individuals will report less positive and more negative experiences than non-lonely individuals will. Finally, we assume the belief in conspiracy theories will be independent of individuals' feelings of loneliness.

## 2. Methods

### 2.1. Participants and procedure

We conducted an anonymous online survey using the Siuvo platform (https://www.siuvo.com) for psychological assessments in healthcare settings between May 2020 and May 2022 in Germany. The survey was distributed using a QR code shared primarily through social media, advertisements, and newsletters. We recruited participants who were aged 18 years and above and gave informed consent. We collected our data conveniently by recruiting populations who had access to the Internet. We used G^*^Power Version 3.1.9.6 to determine sample size. According to a previous study in Germany (50), 32% of people reported being lonely in Germany under the first nationwide lockdown in March 2020. We calculated a sample size of 1,434, which gives an α error rate of 5%, power of 90%, a “small” effect size (*d* = 0.2), and an allocation ratio of 0.32. Considering a 20% dropout rate, 1,721 participants were set as the target sample size. We collected socio-demographic data (i.e., sex, age, and years of education), used a short form of the UCLA Loneliness Scale (ULS-8) to assess feelings of loneliness, as well as the COVID-19 Peritraumatic Distress Instrument (CPDI) and asked individuals' positive and negative experiences of living in the COVID-19 pandemic. We also asked our participants if they have less contact with their family amid COVID-19 and if they have been diagnosed with a mental illness in the past 3 months. The Ethics Committee of Charité—Universitätsmedizin Berlin (EA2/143/20) approved the study.

### 2.2. Measurement of loneliness and perceived positive and negative experiences amid COVID-19 and COVID-19-related distress

Loneliness was assessed by using the well-established short eight-item UCLA Loneliness Scale (ULS-8) in a validated German version (39, 51, 52). Each item was answered on a 4-point Likert scale with total scores ranging from 8 to 32, with higher scores suggesting a higher degree of loneliness. Participants who reported at least sometimes (a cut-off score ≥16) to always feeling lonely were considered “lonely people” (53). We used the 24-item COVID-19 Peritraumatic Distress Index (CPDI) questionnaire to capture peritraumatic psychological distress in the general population amid COVID-19 (54). Each item was answered on a 5-point Likert scale, with higher scores suggesting a higher psychological distress level (a score between 28 and 51 indicates mild-to-moderate distress). Perceived positive experiences during COVID-19 were assessed with a nominal scale by asking, “Do you perceive any positive experiences of the COVID-19 pandemic?” Participants had to choose one of the nine following categorical statements compared to before COVID-19: (1) “No positive effects at all,” (2) “A more environmentally friendly world,” (3) “Time to think about life,” (4) “More time for loved ones,” (5) “More time for hobbies,” (6) “Less work-related stress,” (7) “Less social pressure,” (8) “An increased sense of togetherness,” and (9) “Other positive experiences.” Perceived negative experiences during the pandemic were assessed with a nominal scale by asking, “Did you perceive any negative experiences of the COVID-19 pandemic?” Participants had to choose one of the five following categorical statements that made them feel the most psychological distressed in times of COVID-19: (1) “Corona *per se* is a dangerous infectious disease,” (2) “The epidemic was deliberately manufactured to serve the interest of powerful forces,” (3) “A feeling of imminent threat,” (4) “An edge and worries in general,” and (5) “Other negative experiences.” The items on positive and negative experiences were based on previous studies on life-changing experiences before COVID-19 (e.g., the outbreak of Ebola virus disease, the 2003 SARS epidemic) (55–57) and public views about COVID-19 in Germany (58–61).

### 2.3. Data analysis

We performed statistical analysis by using R Statistical Software (version 4.1.2; R Foundation for Statistical Computing, Vienna, Austria). Differences were considered statistically significant at *p* < 0.05 and highly statistically significant at *p* < 0.01. To check the influences of socio-demographic factors (i.e., sex, age, and years of education) on the loneliness scores, we conducted a multiple linear regression analysis. We used the chi-square test to evaluate if there are differences between non-lonely and lonely people in reporting their contact with families and having been diagnosed with a mental illness in the past 3 months. We used independent sample *t*-tests to examine differences in CPDI scores in non-lonely and lonely people as well as in men and women. We used logistic regression analysis to examine whether there was a significant difference between non-lonely and lonely people in choosing each category of positive and negative experiences. We used the chi-square test to compare men and women and investigate the effects of gender. Finally, we performed logistical regression analyses to understand whether gender, age, and loneliness played a role in conspiracy beliefs.

## 3. Results

### 3.1. Group description

A total of 4,226 participants got access to our survey, 2,466 participants responded to it, 1,858 participants completed it, and 100 participants did not meet the data quality control. Our final sample consists of 1,758 participants (1,304 women (74.2%), age range: 18–75, M = 33.37, SD = 12.24), as shown in Table 1. Each participant completed the survey only once. Of note, 1,354 (77.02%) participants were categorized as “lonely,” as they reported feeling lonely at least sometimes. Our regression analysis revealed that people with less years of education [*t*_(3, 1, 754)_ = −2.16, *p* = 0.03] reported higher loneliness scores. Younger age [*t*_(3, 1, 754)_ = −1.73, *p* = 0.08] and gender did not play a role in the feeling of loneliness [*t*_(3, 1, 754)_ = 1.63, *p* = 0.1]. The the chi-square tests showed that lonely people responded to having less family contact amid COVID-19 as compared to non-lonely people [$\chi_{\left(4\right)}^{2}$ = 177.9, *p* = < 0.001], and there was no significantly higher proportion of lonely people who were diagnosed with a mental illness as compared to non-lonely people [$\chi_{\left(1\right)}^{2}$ = 3.84, *p* = 0.0501]. There were gender differences in reporting psychological distress: CPDI scores were significantly higher in women than men (*p* < 0.001), indicating that women had higher COVID-19-related distress than men.

**Table 1:** Descriptive differences between non-lonely and lonely individuals.

	**All**	**Non-lonely**	**Lonely**	***p***
	***N* = 1,758**	***N* = 404**	***N* = 1,354**	
**Gender**, ***n*** **(%)**				
Women	1,304 (74.2)	282 (69.8)	1,022 (75.5)	0.026
Men	454 (25.8)	122 (30.2)	332 (24.5)	
**Age, mean (SD)**	33.4 (12.2)	35.3 (12.9)	32.8 (12.0)	0.001
**Years of education, mean (SD)**	15.8 (3.68)	16.0 (3.84)	15.8 (3.64)	0.439
**Less family contact**, ***n*** **(%)**				
No	893 (50.8)	321 (79.5)	572 (42.2)	< 0.001
**Prior diagnosis**, ***n*** **(%)**				
Yes	251 (14.3)	46 (11.4)	205 (15.1)	0.0501
**CPDI score, mean (SD)**	35.9 (18.7)	19.1 (13.2)	40.9 (17.1)	< 0.001
**ULS-8 score, mean (SD)**	20.0 (5.83)	12.0 (2.34)	22.4 (4.16)	0.000

### 3.2. Less positive experiences of living in COVID-19 among lonely individuals

**Figure 1:**
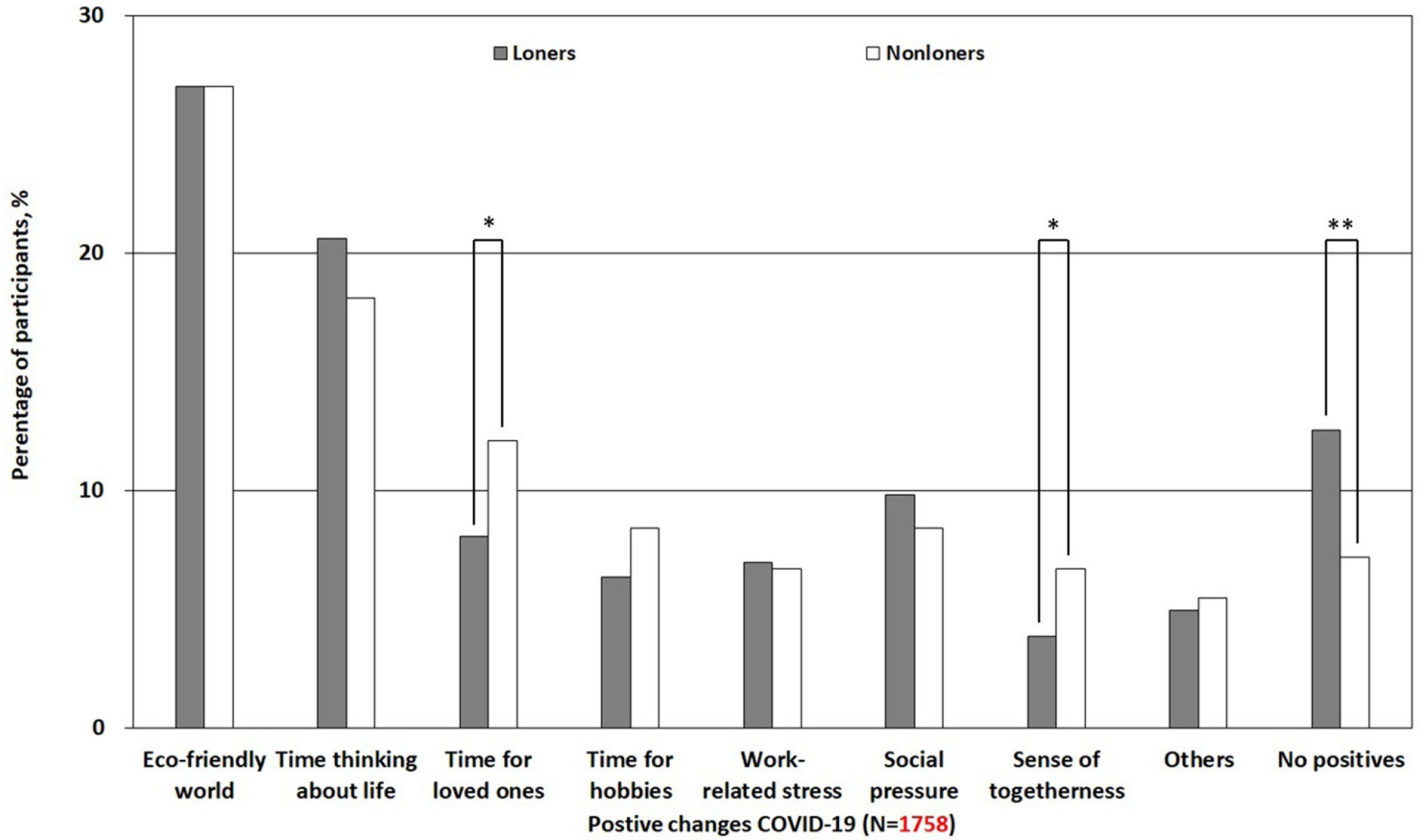
Differences in positive experiences of living in the COVID-19 pandemic among lonely and non-lonely individuals. ^*^p < 0.05; ^**^p < 0.01.

We found that lonely people were more likely to report no positive experience of living during the COVID-19 pandemic as compared to non-lonely people [*z*_(1, 756)_ = 2.92, *p* = 0.004], as shown in Figure 1. In our sample, due to a higher proportion of women, we also conducted additional analyses of men and women separately. In general, we did not find a significant difference between men and women in perceiving no positive experiences during the pandemic [$\chi_{\left(1\right)}^{2}$ = 1.4, *p* = 0.24]. However, we found lonely women were more likely to report no positive experience of living during the COVID-19 pandemic as compared to non-lonely women (*p* = 0.007). In lonely men, we did not find that they were more likely to report no positive experience of living during the COVID-19 pandemic as compared to non-lonely men (*p* = 0.85). Furthermore, lonely people experienced significantly less time with loved ones [*z*_(1, 756)_ = −2.5, *p* = 0.012] and less sense of togetherness [*z*_(1, 756)_ = −2.39, *p* = 0.017] than non-lonely people. There was no significant difference between lonely and non-lonely individuals in experiencing a more “eco-friendly world” [*z*_(1, 756)_ = −0.009, *p* = 0.99], “more time to think about life” [*z*_(1, 756)_ = 1.12, *p* = 0.295], “more time for hobbies” [*z*_(1, 756)_ = −1.44, *p* = 0.15], “less work-related stress” [*z*_(1, 756)_ = 0.181, *p* = 0.857], and “less social pressure” [*z*_(1, 756)_ = 0.846, *p* = 0.398] as positive outcomes of COVID-19.

### 3.3. More negative experiences of living in COVID-19 among lonely individuals

**Figure 2:**
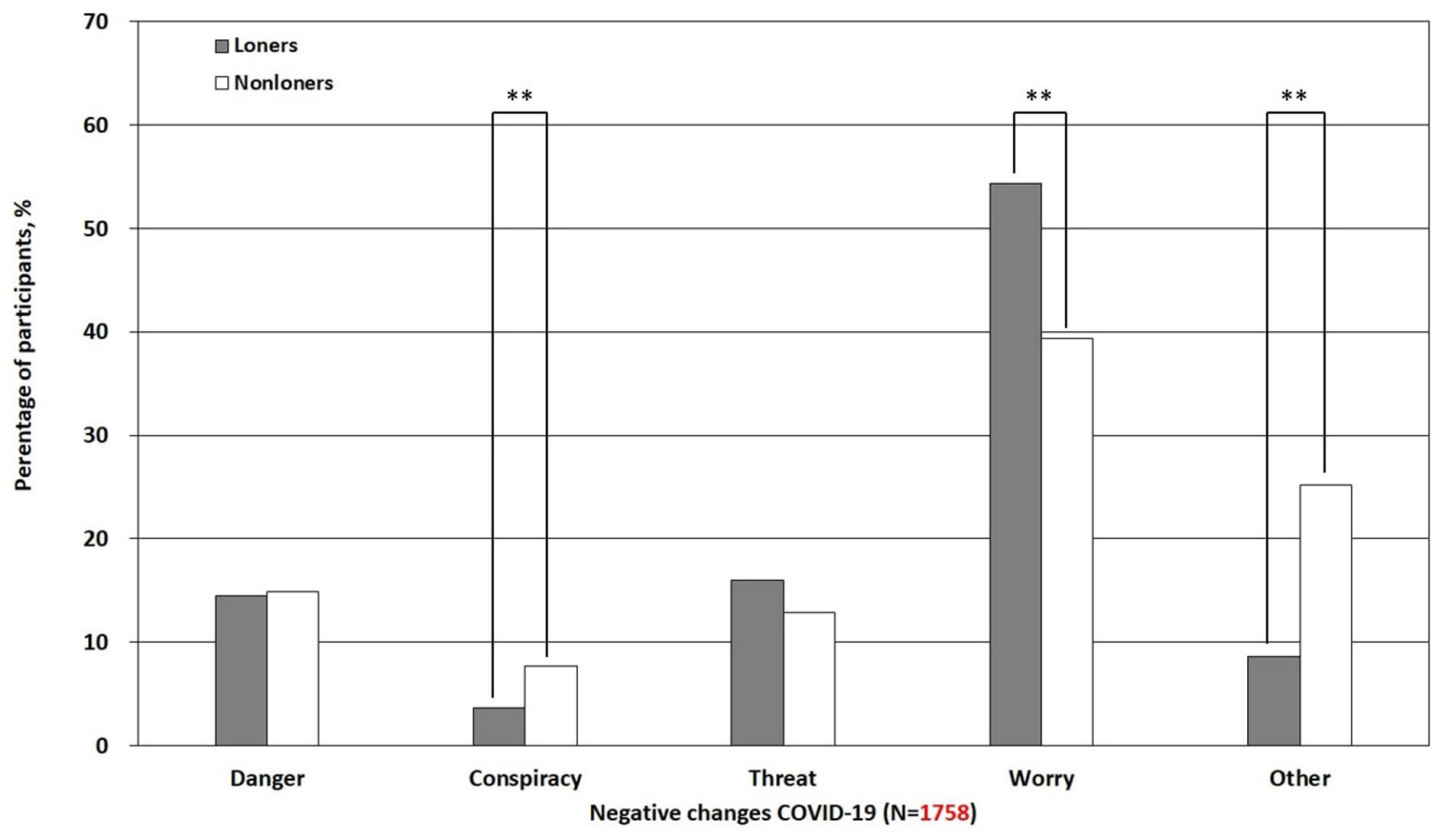
Differences in negative experiences of living in the COVID-19 pandemic among lonely and non-lonely individuals. ^**^p < 0.01.

Lonely participants reported “worry” significantly more often than non-lonely participants [*z*_(1, 756)_ = 6.31, *p* < 0.001], as shown in Figure 2. There was no significant difference between lonely and non-lonely individuals in experiencing “danger” [*z*_(1, 756)_ = −0.19, *p* = 0.85], as well as “threat” [*z*_(1, 756)_ = 1.51, *p* = 0.13]. Non-lonely participants experienced “other” negative outcomes of COVID-19 [*z*_(1, 756)_ = −8.54, *p* < 0.001] significantly more often than lonely participants. The detailed logistical regression results are reported in Table 2. When comparing men with women, we found that women reported worry as a negative experience more often [$\chi_{\left(1\right)}^{2}$ = 7.29, *p* = 0.007] than men. Conversely, men reported perceiving the pandemic as dangerous more often as a negative outcome [$\chi_{\left(1\right)}^{2}$ = 5.29, *p* = 0.007] than women. We did not find a significant difference between men and women in having conspiracy beliefs [$\chi_{\left(1\right)}^{2}$ = 3.68, *p* = 0.055] and perceiving the pandemic as threatening [$\chi_{\left(1\right)}^{2}$ = 0.11, *p* = 0.74]. Overall, our logistic regression showed that age [*z*_(1, 756)_ = 0.15, *p* = 0.88] and gender [*z*_(1, 756)_ = −1.9, *p* = 0.057] were not associated with conspiracy belief, whereas loneliness score was associated with conspiracy belief [*z*_(1, 756)_ = −3.35, *p* < 0.001].

**Table 2:** Differences in positive and negative experiences of living in the COVID-19 pandemic among lonely and non-lonely individuals.

	**All**	**Non-lonely**	**Lonely**	***p***
	***N* = 1,758**	***N* = 404**	***N* = 1,354**	
**No positives**				0.004
Not chosen	1,560 (88.7%)	375 (92.8%)	1,185 (87.5%)	
Chosen	198 (11.3%)	29 (7.18%)	169 (12.5%)	
**Eco-friendly world**				1.000
Not chosen	1,284 (73.0%)	295 (73.0%)	989 (73.0%)	
Chosen	474 (27.0%)	109 (27.0%)	365 (27.0%)	
**Thinking about life**				0.295
Not chosen	1,406 (80.0%)	331 (81.9%)	1,075 (79.4%)	
Chosen	352 (20.0%)	73 (18.1%)	279 (20.6%)	
**Time loved ones**				0.016
Not chosen	1,600 (91.0%)	355 (87.9%)	1,245 (91.9%)	
Chosen	158 (8.99%)	49 (12.1%)	109 (8.05%)	
**Time hobbies**				0.183
Not chosen	1,638 (93.2%)	370 (91.6%)	1,268 (93.6%)	
Chosen	120 (6.83%)	34 (8.42%)	86 (6.35%)	
**Less work-stress**				0.945
Not chosen	1,637 (93.1%)	377 (93.3%)	1,260 (93.1%)	
Chosen	121 (6.88%)	27 (6.68%)	94 (6.94%)	
**Less social pressure**				0.453
Not chosen	1,591 (90.5%)	370 (91.6%)	1,221 (90.2%)	
Chosen	167 (9.50%)	34 (8.42%)	133 (9.82%)	
**Sense of togetherness**				0.022
Not chosen	1,679 (95.5%)	377 (93.3%)	1,302 (96.2%)	
Chosen	79 (4.49%)	27 (6.68%)	52 (3.84%)	
**Other positives**				0.787
Not chosen	1,669 (94.9%)	382 (94.6%)	1,287 (95.1%)	
Chosen	89 (5.06%)	22 (5.45%)	67 (4.95%)	
**Danger**				0.914
Not chosen	1,502 (85.4%)	344 (85.1%)	1,158 (85.5%)	
Chosen	256 (14.6%)	60 (14.9%)	196 (14.5%)	
**Conspiracy**				0.001
Not chosen	1,678 (95.4%)	373 (92.3%)	1,305 (96.4%)	
Chosen	80 (4.55%)	31 (7.67%)	49 (3.62%)	
**Threat**				0.152
Not chosen	1,490 (84.8%)	352 (87.1%)	1,138 (84.0%)	
Chosen	268 (15.2%)	52 (12.9%)	216 (16.0%)	
**Worry**				< 0.001
Not chosen	822 (46.8%)	245 (60.6%)	577 (42.6%)	
Chosen	936 (53.2%)	159 (39.4%)	777 (57.4%)	
**Other negatives**				< 0.001
Not chosen	1,540 (87.6%)	302 (74.8%)	1,238 (91.4%)	
Chosen	218 (12.4%)	102 (25.2%)	116 (8.57%)	

## 4. Discussion

We conducted a cross-sectional study from May 2020 to May 2022 to discover whether there are different positive and negative experiences of living during the COVID-19 pandemic among lonely and non-lonely people. We found less positive and more negative experiences among lonely people as compared with non-lonely people.

Regarding positive experiences, lonely people reported perceiving no positive experiences of living during the pandemic more than non-lonely people. In line with previous studies, lonely people tend to have negative feelings more often (62). Negative feelings and thinking can hint at a generally depressed mood in lonely people, as loneliness can be a sign of depression (63). The negative effect of a depressed state can lead people to withdraw from social life even more (64, 65), which can be an alarming situation during an already-implemented lockdown. In line with Wong's Existential Positive Psychology theory (17), positives cannot exist apart from negatives. Wong conceptualizes wellbeing not only as pursuing positive goals but also as overcoming and mastering negative experiences (17). The absence of positive experiences being especially prominent in lonely people may reflect that they desire ways to deal with negativity and savor positive experiences. In addition to the positive experiences among non-lonely and lonely people, we found that lonely women were more likely to report no positive experience of living during the COVID-19 pandemic as compared to non-lonely women. There was no such a significant difference between non-lonely and lonely men. Further research is needed to examine different positive and negative experiences among non-lonely and lonely men and women.

Furthermore, we found that lonely people perceived “spending more time with loved ones” significantly less than non-lonely people did. It is unclear whether such a perception plays a role in developing and maintaining a desired social relationship that is associated with defying loneliness: Did people not spend enough time with their loved ones and then became lonely, or were they lonely and withdrew themselves even more from social contacts? Another interesting aspect would be to exhibit if lonely people actually spend less time with loved ones than non-lonely people or if they just perceive it as such, as lonely people tend to have more negative and less satisfying social interactions, which contribute to negative moods and interactions (66). In this context, we found that lonely people reported less sense of togetherness than non-lonely people. A sense of togetherness reflects solidarity that comes hand in hand with achieving a common goal (67). Interestingly, loneliness as “selfish” mode can inhibit our sense of solidarity (43, 68, 69). The ETL suggests this “selfish” mode as a response to avoid a dangerous situation (43). However, people may be at risk of developing chronic loneliness, if they continue with this mode over time (70). Loss of sense of togetherness may reinforce this vicious circle. Laitinen and Pessi suggested that chaos and conflict in societies can oppose solidarity, along with the maximization of self-interest (71). Furthermore, our findings uncovered various aspects of what it feels like to be lonely amid COVID-19. Although closer friendships seem to remain intact in the pandemic (72), it is associated with less general peer-to-peer contact (73) and worse recognition of one's own and others' emotions (74), which can lead to more distress and loneliness (73). When looking back at the possible evolutionary functions of loneliness, solidarity and spending time with a peer group help increase social skills for battling loneliness, which in turn lessens their risk for mortality (43). The absence of those social experiences and their effects on the mind and body can be a particular danger for lonely people.

Regarding the negative experiences of living during the pandemic, our results were consistent with the previous studies showing an association between loneliness and worry (75). In our study, lonely people worried significantly more than non-lonely people did. Strikingly, nearly two times as many of the non-lonely individuals believed that the whole pandemic was a hoax and made up of secret powers compared to lonely people. Recent research explains what draws people to conspiracy beliefs. Reasons to believe in conspiracy theories are that they give self-contained explanations for insecure situations, make people feel special, and give them a positive self-image for knowing information that nobody else knows and/or satisfy people's needs for safety and security (76). Other important socio-demographic data related to conspiracy beliefs are socio-economic status, education and political positions (76). Finally, our results showed that non-lonely individuals experienced more “other” negative outcomes of COVID-19 than lonely participants did. Negative experiences that were not mentioned before may include, among other factors, prolonged grievances for loved ones who died (77–79), financial hardships (80, 81), and family problems (82, 83).

Generalizability and representativeness are limited in our study populations (e.g., people 18 ± 75 years old), although our socio-demographic characteristics are consistent with other published surveys during COVID-19 (84, 85). To reduce sampling bias, respondents were from Germany's 16 federal states and worked in various fields, such as office administration, healthcare, education, civil service, sales, agriculture, arts, sports, and media. Moreover, our sample consists of a high rate of women, which is consistent with other published surveys during COVID-19 (84, 85). Our findings cannot establish a cause-and-effect relationship or analyze momentary experiences and daily behavior over a period. We cannot rule out the varying influences of COVID-19 and its related measures (e.g., lockdowns, public health, social, and economic measures) on our observed results. Future studies may also consider different socio-demographic factors (e.g., marital status, migration background, and household) that may have influences on the observed effects. A depth quality assessment is needed to uncover other potential positive and negative experiences with the COVID-19 pandemic.

## 5. Conclusion

The COVID-19 crisis may be a chance to reconsider personal and social priorities. Our study showed that lonely people reported more negative and less positive experiences during the COVID-19 pandemic than non-lonely people. To prepare for and respond to future pandemics, decision-makers may seek to spread positive energy among vulnerable populations (e.g., people who feel lonely), build trust and confidence in dealing with negative experiences (e.g., worry and fear), and improve communication. Future studies may focus on effective, adaptive, and scalable interventions to foster social cohesion, empower people helps build resilience, and, most importantly, provide timely social care.

## Data availability statement

The datasets are available, with restrictions, due to confidentiality in line with Berlin Data Protection Act (Berliner Datenschutzgesetz - BlnDSG). Interested individuals can contact stresshealth@charite.de.

## Ethics statement

The studies involving human participants were reviewed and approved by Ethics Committee of Charité – Universitätsmedizin Berlin. The participants provided their written informed consent to participate in this study.

## Author contributions

Conceptualization, writing—review and editing, and supervision: SL. Methodology, data collection, and data curation: LW and SL. Data analysis and writing—original draft preparation: LW. Both authors have read and agreed to the published version of the manuscript.

## References

[1] Kerekes S Badea AG-E Paun D. Analyzing lockdown policies and their effectiveness in Romania and Hungary. Challenges. (2021) 12:20. 10.3390/challe12020020

[2] Amerio A Aguglia A Odone A Gianfredi V Serafini G Signorelli C. Covid-19 pandemic impact on mental health of vulnerable populations. Acta Biomed. (2020) 91:95–6. 10.23750/abm.v91i9-S.10112 32701924 PMC8023095

[3] Siegel RM Mallow PJ. The impact of COVID-19 on vulnerable populations and implications for children and health care policy. Clin Pediatr. (2021) 60:93–8. 10.1177/0009922820973018 33243000

[4] Webber-Ritchey KJ Simonovich SD Spurlark RS. COVID-19: qualitative research with vulnerable populations. Nurs Sci Quart. (2021) 34:13–9. 10.1177/0894318420965225 33349176

[5] Brown G Greenfield PM. Staying connected during stay-at-home: communication with family and friends and its association with well-being. Hum Behav Emerg Technol. (2021) 3:147–56. 10.1002/hbe2.246

[6] Nguyen MH Gruber J Marler W Hunsaker A Fuchs J Hargittai E. Staying connected while physically apart: digital communication when face-to-face interactions are limited. N Media Soc. (2022) 24:2046–67. 10.1177/1461444820985442

[7] Khan I Shah D Shah SS. COVID-19 pandemic and its positive impacts on environment: an updated review. Int J Environ Sci Technol. (2021) 18:521–30. 10.1007/s13762-020-03021-3 33224247 PMC7668666

[8] World Health Organization. COVID-19 Pandemic Triggers 25% Increase in Prevalence of Anxiety and Depression Worldwide. World Health Organization. Available online at: https://www.who.int/news/item/02–03-2022-covid-19-pandemic-triggers-25-increase-in-prevalence-of-anxiety-and-depression-worldwide (accessed December 06, 2022).

[9] Uddin M. Addressing work-life balance challenges of working women during COVID-19 in Bangladesh. Int Soc Sci J. (2021) 71:7–20. 10.1111/issj.12267 34230685 PMC8251227

[10] Sarrasanti N Donkor FK Santos C Tsagkari M Wannous C. Its about time we care about an equitable world: women's unpaid care work and COVID-19. IEEE Eng Manag Rev. (2020) 48:37–45. 10.1109/EMR.2020.3031313

[11] Kalabikhina IE. Demographic and social issues of the pandemic. Popul Econ. (2020) 4:103–22. 10.3897/popecon.4.e53891

[12] Agostinelli F Doepke M Sorrenti G Zilibotti F. When the great equalizer shuts down: schools, peers, and parents in pandemic times. J Public Econ. (2022) 206:104574. 10.1016/j.jpubeco.2021.104574 35017763 PMC8735857

[13] Bettinger-Lopez C Bro A. A double pandemic: domestic violence in the age of COVID-19. Council Foreign Relat. (2020) 13.

[14] Navarro V. The consequences of neoliberalism in the current pandemic. Int J Health Serv. (2020) 50:271–5. 10.1177/0020731420925449 32380877 PMC7218352

[15] Rosenfeld DL Tomiyama AJ. Can a pandemic make people more socially conservative? Political ideology, gender roles, and the case of COVID-19. J Appl Soc Psychol. (2021) 51:425–33. 10.1111/jasp.12745 33821034 PMC8014651

[16] Wong P. Courage, Faith, Meaning, Mature Happiness in Dangerous Times. (2017). Available online at: http://www.drpaulwong.com/inpm-presidents-report-may-2017/ (accessed December 06, 2022).

[17] Wong PT Mayer C-H Arslan G. COVID-19 and existential positive psychology (PP2. 0): the new science of self-transcendence. Front Psychol. (2021) 12:800308. 10.3389/fpsyg.2021.800308 34956025 PMC8699172

[18] Novaković IZ Lueger-Schuster B Verginer L Bakić H Ajduković D Borges C. You can't do anything about it, but you can make the best of it: a qualitative analysis of pandemic-related experiences in six European countries. Eur J Psychotraumatol. (2022) 13:2065431. 10.1080/20008198.2022.2065431 35646295 PMC9132427

[19] Bates CR Nicholson LM Rea EM Hagy HA Bohnert AM. Life interrupted: family routines buffer stress during the COVID-19 pandemic. J Child Fam Stud. (2021) 30:2641–51. 10.1007/s10826-021-02063-6 34404970 PMC8360776

[20] Wright L Fluharty M Steptoe A Fancourt D. How did people cope during the COVID-19 pandemic? A structural topic modelling analysis of free-text data From 11,000 United Kingdom adults. Front Psychol. (2022) 13:810655. 10.3389/fpsyg.2022.810655 35734451 PMC9207408

[21] Ipsen C van Veldhoven M Kirchner K Hansen JP. Six key advantages and disadvantages of working from home in Europe during COVID-19. Int J Environ Res Public Health. (2021) 18:1826. 10.3390/ijerph18041826 33668505 PMC7917590

[22] Büssing A Rodrigues Recchia D Hein R Dienberg T. Perceived changes of specific attitudes, perceptions and behaviors during the corona pandemic and their relation to wellbeing. Health Qual Life Outcomes. (2020) 18:1–17. 10.1186/s12955-020-01623-6 33256755 PMC7702679

[23] Wong PT Arslan G Bowers VL Peacock EJ Kjell ONE Ivtzan I. Self-transcendence as a buffer against COVID-19 suffering: the development and validation of the self-transcendence measure-B. Front Psychol. (2021) 12:648549. 10.3389/fpsyg.2021.648549 34690853 PMC8527188

[24] Cénat JM Farahi SMMM Dalexis RD Darius WP Bekarkhanechi FM Poisson H. The global evolution of mental health problems during the COVID-19 pandemic: A systematic review and meta-analysis of longitudinal studies. J Affect Disord. (2022) 315:70–95. 10.1016/j.jad.2022.07.011 35842064 PMC9278995

[25] Brooks SK Webster RK Smith LE Woodland L Wessely S Greenberg N. The psychological impact of quarantine and how to reduce it: rapid review of the evidence. Lancet. (2020) 395:912–20. 10.1016/S0140-6736(20)30460-8 32112714 PMC7158942

[26] Haucke M Liu S Heinzel S. The persistence of the impact of COVID-19–related distress, mood inertia, and loneliness on mental health during a post-lockdown period in Germany: an ecological momentary assessment study. JMIR Mental Health. (2021) 8:e29419. 10.2196/29419 34347622 PMC8396535

[27] Zhang SX Batra K Xu W Liu T Dong RK Yin A. Mental disorder symptoms during the COVID-19 pandemic in Latin America–a systematic review and meta-analysis. Epidemiol Psychiatr Sci. (2022) 31:e23 10.1017/S2045796021000767 35438066 PMC9069590

[28] Zhang SX Chen J. Scientific evidence on mental health in key regions under the COVID-19 pandemic–meta-analytical evidence from Africa, Asia, China, Eastern Europe, Latin America, South Asia, Southeast Asia, and Spain. Eur J Psychotraumatol. (2021) 12:2001192. 10.1080/20008198.2021.2001192 34900123 PMC8654399

[29] Islam MR Quaiyum S Pakhe SA Repon MAU Bhuiyan MA. Dataset concerning the mental health of healthcare professionals during COVID-19 pandemic in Bangladesh. Data Brief . (2021) 39:107506. 10.1016/j.dib.2021.107506 34729387 PMC8553544

[30] Zysberg L Zisberg A. Days of worry: emotional intelligence and social support mediate worry in the COVID-19 pandemic. J Health Psychol. (2022) 27:268–77. 10.1177/1359105320949935 32811195

[31] Nam S-H Nam J-H Kwon C-Y. Comparison of the mental health impact of COVID-19 on vulnerable and non-vulnerable groups: a systematic review and meta-analysis of observational studies. Int J Environ Res Public Health. (2021) 18:10830. 10.3390/ijerph182010830 34682574 PMC8535316

[32] Attema AE L'haridon O Raude J Seror V Group C. Beliefs and risk perceptions about COVID-19: evidence from two successive French representative surveys during lockdown. Front Psychol. (2021) 12:619145. 10.3389/fpsyg.2021.619145 33597909 PMC7882490

[33] Akbari M Spada MM Nikčević AV Zamani E. The relationship between fear of COVID-19 and health anxiety among families with COVID-19 infected: the mediating role of metacognitions, intolerance of uncertainty and emotion regulation. Clin Psychol Psychother. (2021) 28:1354–66. 10.1002/cpp.2628 34110670

[34] Schuller S. World conspiracy literature and antisemitism. Transit. (2021) 13:195–204. 10.5070/T713153441

[35] Haucke M Heinz A Liu S Heinzel S. The impact of COVID-19 lockdown on daily activities, cognitions, and stress in a lonely and distressed population: temporal dynamic network analysis. J Med Internet Res. (2022) 24:e32598. 10.2196/32598 35191843 PMC8972118

[36] Jeste DV Lee EE Cacioppo S. Battling the modern behavioral epidemic of loneliness: suggestions for research and interventions. JAMA Psychiatry. (2020) 77:553–4. 10.1001/jamapsychiatry.2020.0027 32129811 PMC7483387

[37] Krieger T Seewer N. Einsamkeit / Tobias Krieger, Noëmi Seewer. Göttingen: Hogrefe (2022). 10.1026/03172-000

[38] Cacioppo JT Cacioppo S Boomsma DI. Evolutionary mechanisms for loneliness. Cogn Emot. (2014) 28:3–21. 10.1080/02699931.2013.837379 24067110 PMC3855545

[39] Liu S Haucke MN Heinzel S Heinz A. Long-term impact of economic downturn and loneliness on psychological distress: triple crises of COVID-19 pandemic. J Clin Med. (2021) 10:4596. 10.3390/jcm10194596 34640614 PMC8509467

[40] Ernst M Niederer D Werner AM Czaja SJ Mikton C Ong AD. Loneliness before and during the COVID-19 pandemic: a systematic review with meta-analysis. Am Psychol. (2022) 77: 660–77. 10.1037/amp0001005 35533109 PMC9768682

[41] Bello UM Kannan P Chutiyami M Salihu D Cheong AM Miller T. Prevalence of anxiety and depression among the general population in Africa during the COVID-19 pandemic: a systematic review and meta-Analysis. Front Public Health. (2022) 10:814981. 10.3389/fpubh.2022.814981 35655463 PMC9152218

[42] Johns G Samuel V Freemantle L Lewis J Waddington L. The global prevalence of depression and anxiety among doctors during the covid-19 pandemic: systematic review and meta-analysis. J Affect Disord. (2022) 298:431–41. 10.1016/j.jad.2021.11.026 34785264 PMC8596335

[43] Cacioppo JT Cacioppo S. Loneliness in the modern age: an evolutionary theory of loneliness (ETL). Adv Exp Soc Psychol. (2018) 58: 27–97. 10.1016/bs.aesp.2018.03.003

[44] Spithoven AW Bijttebier P Goossens L. It is all in their mind: a review on information processing bias in lonely individuals. Clin Psychol Rev. (2017) 58:97–114. 10.1016/j.cpr.2017.10.003 29102150

[45] Bellucci G. Positive attitudes and negative expectations in lonely individuals. Scientific Reports. (2020) 10:1–9. 10.1038/s41598-020-75712-3 33122843 PMC7596507

[46] Lamster F Nittel C Rief W Mehl S Lincoln T. The impact of loneliness on paranoia: an experimental approach. J Behav Ther Exp Psychiatry. (2017) 54:51–7. 10.1016/j.jbtep.2016.06.005 27362838

[47] Suthaharan P Reed EJ Leptourgos P Kenney JG Uddenberg S Mathys CD. Paranoia and belief updating during the COVID-19 crisis. Nat Hum Behav. (2021) 5:1190–202. 10.1038/s41562-021-01176-8 34316049 PMC8458246

[48] Douglas KM Sutton RM. Does it take one to know one? Endorsement of conspiracy theories Is influenced by personal willingness to conspire. Br J Soc Psychol. (2011) 50:544–52. 10.1111/j.2044-8309.2010.02018.x 21486312

[49] Dow BJ Johnson AL Wang CS Whitson J Menon T. The COVID-19 pandemic and the search for structure: social media and conspiracy theories. Soc Pers Psychol Compass. (2021) 15:e12636. 10.1111/spc3.12636 34512798 PMC8420120

[50] Berger K Riedel-Heller S Pabst A Rietschel M Richter D. [Loneliness during the first wave of the SARS-CoV-2 pandemic-results of the German National Cohort (NAKO)]. Bundesgesundheitsblatt Gesundheitsforschung Gesundheitsschutz. (2021) 64:1157–64. 10.1007/s00103-021-03393-y 34327541 PMC8320420

[51] Döring N Bortz J. Psychometrische Einsamkeitsforschung: Deutsche Neukonstruktion der UCLA Loneliness Scale [Psychometric research on loneliness: A new German version of the University of California at Los Angeles (UCLA) Loneliness Scale]. Diagnostica. (1993) 39:224–39.

[52] Liu S Heinzel S Haucke MN Heinz A. Increased psychological distress, loneliness, and unemployment in the spread of COVID-19 over 6 months in Germany. Medicina. (2021) 57:53. 10.3390/medicina57010053 33435368 PMC7827929

[53] Hays RD DiMatteo MR. A short-form measure of loneliness. J Pers Assess. (1987) 51:69–81. 10.1207/s15327752jpa5101_6 3572711

[54] Qiu J Shen B Zhao M Wang Z Xie B Xu Y. A nationwide survey of psychological distress among Chinese people in the COVID-19 epidemic: implications and policy recommendations. Gen Psychiatry. (2020) 33:e100213. 10.1136/gpsych-2020-100213 32215365 PMC7061893

[55] Thompson RR Garfin DR Holman EA Silver RC. Distress, worry, and functioning following a global health crisis: a national study of Americans' responses to Ebola. Clin Psychol Sci. (2017) 5:513–21. 10.1177/2167702617692030

[56] Fredrickson BL Tugade MM Waugh CE Larkin GR. What good are positive emotions in crisis? A prospective study of resilience and emotions following the terrorist attacks on the United States on September 11th, 2001. J Pers Soc Psychol. (2003) 84:365. 10.1037/0022-3514.84.2.365 12585810 PMC2755263

[57] Lau JT Yang X Pang E Tsui H Wong E Wing YK. SARS-related perceptions in Hong Kong. Emerg Infect Dis. (2005) 11:417. 10.3201/eid1103.040675 15757557 PMC3298267

[58] Freudental R. Die positiven Folgen der Corona-Krise: Mehrheit glaubt, Pandemie bringt Familie und Freunde näher zusammen. Available online at: https://www.ipsos.com/de-de/die-positiven-folgen-der-corona-krise-mehrheit-glaubt-pandemie-bringt-familie-und-freunde-naher (accessed December 06, 2022).

[59] Der Spiegel. Coronakrise führt zu historischem CO2-Rückgang. Avaialble online at: https://www.spiegel.de/wirtschaft/unternehmen/corona-krise-fuehrt-zu-historischem-co2-rueckgang-a-66efc1a4-b572-4013-88a0-38b5510a0a73 (accessed December 06, 2022).

[60] Drosner R. Homeoffice in Corona-Zeiten - ≪Viele vermissen die Wertschätzung der Vorgesetzten≫. Available online at: https://www.srf.ch/wissen/corona/homeoffice-in-corona-zeiten-viele-vermissen-die-wertschaetzung-der-vorgesetzten (accessed December 06, 2022).

[61] Naumann E Mata J Reifenscheid M Möhring K Wenz A Rettig T. Die Mannheimer Corona-Studie: Schwerpunktbericht zum Angstempfinden in der Bevölkerung. Survey Results. University of Mannheim (2020). Available online at: https://www.unimannheim.de/media/Einrichtungen/gip/Corona_Studie/Schwerpunktbericht_Angstempfinden_Mannheimer_Corona_Studie.pdf

[62] Coşan D. An evaluation of loneliness. Eur Proc Soc Behav Sci. (2014) 1:103–10. 10.15405/epsbs.2014.05.13

[63] Erzen E Çikrikci Ö. The effect of loneliness on depression: a meta-analysis. International *J Soc Psychiatry*. (2018) 64:427–35. 10.1177/0020764018776349 29792097

[64] Derntl B Seidel E-M Eickhoff SB Kellermann T Gur RC Schneider F. Neural correlates of social approach and withdrawal in patients with major depression. Soc Neurosci. (2011) 6:482–501. 10.1080/17470919.2011.579800 21777105 PMC3203307

[65] Lee JI Parhar IS Soga T. Hikikomori: social withdrawal a risk factor for depression. Psychiatry Clin Neurosci. (2022) 76:340–8. 10.1111/pcn.13354 35338539

[66] Hawkley LC Preacher KJ Cacioppo JT. Multilevel modeling of social interactions and mood in lonely and socially connected individuals: the MacArthur social neuroscience studies. In:Ong AD van Dulmen MHM, editors. Oxford Handbook of Methods in Positive Psychology. Oxford University Press (2007). p. 559–75.

[67] Bayertz K. Four uses of “solidarity”. In:Bayertz K, editor. Solidarity. Dordrecht: Kluwer (1999). p. 3–28. 10.1007/978-94-015-9245-1_1

[68] Swann WB Jr Gómez Á Dovidio JF Hart S Jetten J. Dying and killing for one's group: identity fusion moderates responses to intergroup versions of the trolley problem. Psychol Sci. (2010) 21:1176–83. 10.1177/0956797610376656 20622141

[69] Kramer RM Brewer MB. Effects of group identity on resource use in a simulated commons dilemma. J Pers Soc Psychol. (1984) 46:1044. 10.1037/0022-3514.46.5.1044 6737205

[70] Krieger T Seewer N Skoko A. chronische einsamkeit–mehr als ein symptom einer depression. PiD-Psychotherapie im Dialog. (2021) 22:59–63. 10.1055/a-1215-1677

[71] Laitinen A Pessi AB. “Solidarity: Theory and practice. An introduction,” in Laitinen A, Pessi AB, editors. Solidarity: Theory and Practice. Lanham, MD: Lexington Books (2015), pp. 1–29.

[72] Cook EC. Perceived changes in peer relationships and behavioral health among college students during COVID-19. J Am Coll Health. (2022) 1–8. 10.1080/07448481.2022.2106787 35930457

[73] Song Q Vicman JM Doan SN. Changes in attachment to parents and peers and relations with mental health during the COVID-19 pandemic. Emerg Adulth. (2022) 10:1048–60. 10.1177/21676968221097167 35935716 PMC9260195

[74] Tambelli R Cimino S Marzilli E Ballarotto G Cerniglia L. Late adolescents' attachment to parents and peers and psychological distress resulting from COVID-19. A study on the mediation role of alexithymia. Int J Environ Res Public Health. (2021) 18:10649. 10.3390/ijerph182010649 34682393 PMC8535909

[75] Theeke LA Mallow J Gianni C Legg K Glass C. The experience of older women living with loneliness and chronic conditions in Appalachia. J Rural Mental Health. (2015) 39:61. 10.1037/rmh0000029 26594267 PMC4650889

[76] Douglas KM Uscinski JE Sutton RM Cichocka A Nefes T Ang CS. Understanding conspiracy theories. Polit Psychol. (2019) 40:3–35. 10.1111/pops.12568

[77] Eisma MC Tamminga A Smid GE Boelen PA. Acute grief after deaths due to COVID-19, natural causes and unnatural causes: an empirical comparison. J Affect Disord. (2021) 278:54–6. 10.1016/j.jad.2020.09.049 32950843 PMC7487144

[78] Gang J Falzarano F She WJ Winoker H Prigerson HG. Are deaths from COVID-19 associated with higher rates of prolonged grief disorder (PGD) than deaths from other causes? Death Stud. (2022) 46:1287–96. 10.1080/07481187.2022.2039326 35167429 PMC9254485

[79] Khoury B Barbarin O Gutiérrez G Klicperova M Padakannaya P Thompson A. (2022). Complicated grief during COVID-19: An international perspective. Int Perspect Psychol. 11:214–221. 10.1027/2157-3891/a00005536311343

[80] Cleaveland CL Frankenfeld CL. Household financial hardship factors are strongly associated with poorer Latino mental health during COVID-19. J Racial Ethnic Health Disparit. (2022) 1–14. 10.1007/s40615-022-01366-8 35831704 PMC9281376

[81] Goins RT Anderson E Minick H Daniels H. Older adults in the United States and COVID-19: a qualitative study of perceptions, finances, coping, and emotions. Front Public Health. (2021) 9:660536. 10.3389/fpubh.2021.660536 34504824 PMC8421518

[82] Athapathu A Navaratnam D Doluweera M Liyanage G. Child emotional and behavioral difficulties and parent stress during COVID-19 lockdown in Sri Lankan families. PLoS ONE. (2022) 17:e0271757. 10.1371/journal.pone.0271757 35921371 PMC9348699

[83] Kamoshida S Nihonmatsu N Takagi G Wakashima K. The relationship between family variables and family social problems during the COVID-19 pandemic. PLoS ONE. (2022) 17:e0270210. 10.1371/journal.pone.0270210 35767548 PMC9242492

[84] Öksüz EE Kalkan B Can N Haktanir A. Adult mental health and loneliness during the COVID-19 pandemic in late 2020. Eur J Psychol Open. (2021) 80:1–13. 10.1024/2673-8627/a000001

[85] McQuaid RJ Cox SM Ogunlana A Jaworska N. The burden of loneliness: implications of the social determinants of health during COVID-19. Psychiatry Res. (2021) 296:113648. 10.1016/j.psychres.2020.113648 33348199 PMC9754822

